# Neoaortoiliac reconstruction for a *Histoplasma capsulatum* endograft infection

**DOI:** 10.1016/j.jvscit.2025.102075

**Published:** 2025-11-27

**Authors:** Punit Vyas, Grant Woodruff, Thomas Naslund

**Affiliations:** Vanderbilt University Medical Center, Department of Vascular Surgery, Nashville, TN

**Keywords:** Neoaortoiliac, *Histoplasma*, Endovascular

## Abstract

We present a case of a 64-year-old man with a history of endovascular abdominal aortic aneurysm repair endovascular aneurysm repair in 2015, who developed a graft infection owing to *Histoplasma capsulatum* in 2025. The patient underwent a neoaortoiliac system procedure using autologous femoral veins for reconstruction with continued itraconazole therapy in the outpatient setting. This case underscores the importance of considering fungal pathogens in graft infections and highlights the effectiveness of the neoaortoiliac system procedure in conjunction with antimicrobials in managing such complex cases.

Vascular graft infections (VGIs) remain a feared complication after endovascular aneurysm repair (EVAR), characterized by diagnostic ambiguity, therapeutic complexity, and high associated morbidity and mortality rates. Incidence rates are generally reported between 0.6% and 3.0%.^1^^,^^2^ The presentation of VGIs can be insidious and nonspecific, often delaying diagnosis and treatment. The microbiological landscape of VGIs are typically dominated by bacteria, most commonly *Staphylococcus aureus*, *Pseudomonas Aeruginosa*, and *Escherichia coli*.^3^^,^^4^ Less commonly, enteric bacteria, fungi, and anaerobes are implicated, particularly in cases of aortoenteric fistula.^5^

Fungal VGIs represent a rare and underdiagnosed subset, with even less specific presentations. They are typically associated with immunocompromised states, prolonged antibiotic exposure, or prior fungal colonization. *Candida* species account for most reported fungal cases, with *Aspergillus* and *Histoplasma* being encountered much less frequently.^5^, ^6^, ^7^ This case highlights the course of a patient with *Histoplasma*-related endograft infection and discusses the multidisciplinary strategy that led to successful management.

Consent for publication of this case report was obtained from the patient.

## Case report

A 64-year-old man with past medical history notable for treated latent tuberculosis infection, a history of incarceration in early adulthood, and an active smoking history presented 9 years after prior EVAR for an infrarenal abdominal aortic aneurysm with abdominal pain. The discomfort had been ongoing for the past month and was most prominent in the left lower quadrant, radiating to the left groin. He denied fevers or chills, but reported intermittent night sweats over the past 6 months. He endorsed no weight loss. The prior EVAR was indicated for an asymptomatic 5.5-cm infrarenal abdominal aortic aneurysm with an aneurysmal right common iliac artery measuring 2.4 cm. The aneurysm was repaired with a Gore excluder endograft. On completion angiogram, a type Ia endoleak was identified, and a Gore aortic cuff was used for proximal seal up to the renal arteries.

**Fig 1:**
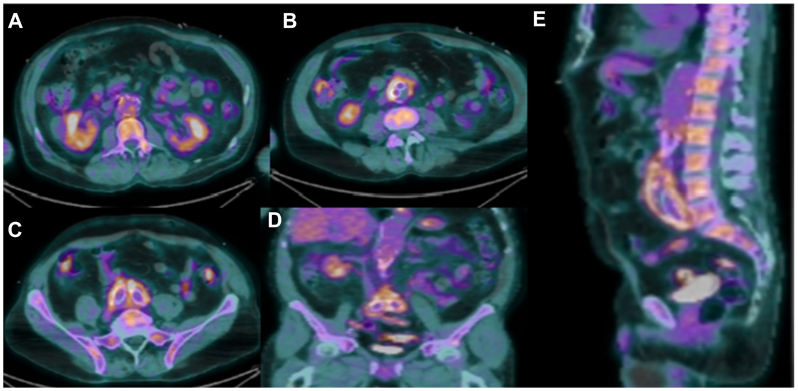
Positron emission tomography with computed tomography (PET-CT) preoperative scan showing fluorodeoxyglucose avidity along the entirety of the graft with adjacent inflammatory stranding. **(A)** Axial image, at the level of the renal arteries. **(B** and **C)** Axial images showing uptake of limbs of the aortic prosthesis. **(D** and **E)** Coronal and sagittal views.

Computed tomography (CT) revealed enlargement of the aneurysm sac to 4.2 × 4.8 cm, which has increased from 3.4 × 2.5 cm 1 year prior, with wall thickening, raising suspicion for a VGI. Laboratory analysis demonstrated a normal leukocyte count, but elevated C-reactive protein (69 mg/L). Empiric antibiotic therapy was initiated with intravenous vancomycin, cefepime, and metronidazole. Positron emission tomography with CT (PET-CT) demonstrated avid fluorodeoxyglucose uptake along the entire length of the aortic endograft with absence of uptake in the other vascular beds making vasculitis a less likely diagnosis, and confirming suspicions for a VGI (Fig 1, *A*-*E*).

The patient was taken to the operating room for explantation of the infected endograft and neoaortoiliac system (NAIS) reconstruction. Femoral veins were harvested from the level of the bifurcation of the common femoral vein to the popliteal vein. The valves were lysed and the veins reversed, spatulated, and joined as a syndactyly to create a pantaloon with a running 5-0 Prolene suture. Next, abdominal exploration identified extensive adhesions throughout the intraperitoneal cavity, likely secondary to inflammatory changes around the aorta. No aortoenteric fistulas were identified. To facilitate excision, the suprarenal aorta was clamped; there was no suprarenal involvement. The infected graft was removed in its entirety along with the surrounding aortic tissue from the external iliac arteries (EIAs) to the aortic neck, nearly 35 cm^2^ of aortic tissue. After excision, the aortic clamp was repositioned infrarenal and the prepared pantaloon graft was sutured end-to-end at the infrarenal aortic neck and end-to-end at the bilateral common iliac artery bifurcations. Balloon occlusion was used to control the EIAs during the anastomosis. No pulse was noted distal to the anastomosis on the left and an EIA dissection was identified, likely from balloon occlusion. An Abbott Absolute stent was placed in the proximal EIA and an Abbott Absolute Pro stent was placed extending coverage to the pelvic brim via access from the superficial femoral artery. Flow was confirmed with palpable pulses and angiography.

The aortic bed was irrigated and debrided using a Pulsavac system (Zimmer Biomet). A vascularized omental pedicle flap was mobilized and circumferentially wrapped around the NAIS conduit to provide vascularized soft tissue coverage.

**Fig 2:**
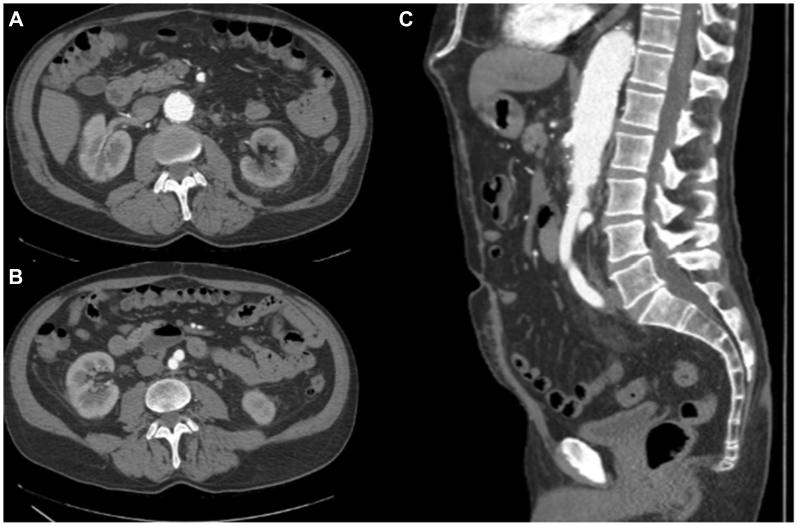
Arterial phase of cross-sectional imaging, 4 months postoperative from neoaortoiliac system (NAIS) procedure. Minimal perigraft edema, without fluid collections showing expected and appropriate postsurgical changes. **(A** and **B)** Axial image. **(C)** Sagittal image.

Postoperatively, the patient was hemodynamically stable, extubated, and had his nasogastric tube removed with return of bowel function. Intravenous antimicrobials, including vancomycin (pharmacy-managed therapeutic dosing), cefepime (2 g every 8 hours), and metronidazole (500 mg every 12 hours) were continued until intraoperative tissue cultures resulted. One week later, intraoperative samples returned *Candida albicans*. The antimicrobial plan transitioned to oral fluconazole (400 mg/day for 4 weeks), doxycycline (100 mg/day for 4 weeks), and metronidazole (500 mg every 12 hours for 2 weeks). Two weeks later, intraoperative cultures from the infected graft resulted positive for *H capsulatum*, and the patient was transitioned to oral itraconazole monotherapy (200 mg/day for 12 months). Respiratory cultures obtained perioperatively as part of an infectious workup revealed *Histoplasma*, suggesting potential pulmonary involvement and dissemination. The patient was treated under infectious disease supervision with itraconazole for a planned duration of 12 months. The 4-month postoperative CT (Fig 2, *A*-*C*) displayed mild and expected postoperative inflammation without evidence of fluid collections or disruptions near the suture lines. The patient has had no clinical signs or symptoms of active infection.

## Discussion

*H capsulatum* is a dimorphic fungus that typically presents as a pulmonary infection, but can disseminate, particularly in immunocompromised patients. In VGIs, *Histoplasma* has been implicated in only a few documented cases, and diagnosis is often delayed owing to its nonspecific clinical presentation.^1^^,^^5^, ^6^, ^7^ In this case, despite negative initial cultures, persistent concern for VGI prompted further imaging with PET-CT scan, explantation, and identification of *Histoplasma* from intraoperative fungal cultures. Notably, the treatment facility and patient's primary residence was within the endemic territory for *Histoplasma*, and the patients' prior history of tuberculosis and smoking are risk factors for developing respiratory or disseminated complications.^7^^,^^8^

The diagnostic challenges of VGIs lie in their subtle clinical manifestations. Patients may present with fevers, malaise, or persistent localized pain, and standard blood cultures frequently fail to yield a pathogen. Imaging studies, such as PET-CT scans, play a crucial role in detecting infection, but diagnosis requires histopathological examination and culture of resected graft material, often in conjunction with serum cultures and markers.^9^ In this case, diagnosis was made postoperatively after intraoperative fungal tissue cultures returned positive for *Histoplasma*, highlighting the importance of comprehensive intraoperative microbiological evaluation. The respiratory culture resulting in *Histoplasma* did not change the management of this VGI, but may provide context for its pathogenesis.

The NAIS procedure has been used increasingly in cases where the explantation of an infected graft is necessary. Studies have demonstrated the durability of femoral vein grafts, with low rates of recurrent infection and graft failure.^1^ For fungal VGIs, appropriate antifungal therapy is essential. Itraconazole is the treatment of choice for *H capsulatum* owing to its efficacy against this organism, especially with consideration of possible pulmonary dissemination, as in this case.^7^^,^^10^^,^^11^

## Conclusions

This case highlights the necessity of considering fungal organisms, including *H capsulatum*, in the differential for VGIs, particularly in patients with risk factors for dissemination of atypical infections. The NAIS procedure serves as an effective treatment modality for managing complex aortic infections, providing a durable and infection-resistant reconstruction option. Given the increasing prevalence of EVAR and the potential for late-onset infections, continued surveillance and early intervention remain crucial in preventing catastrophic complications.

## Funding

None.

## Disclosures

None.
